# Assessing the Potential of Molecular Imaging for Myelin Quantification in Organotypic Cultures

**DOI:** 10.3390/pharmaceutics13070975

**Published:** 2021-06-28

**Authors:** Ander Egimendia, Susana Carregal-Romero, Iñaki Osorio-Querejeta, Daniel Padro, Jesús Ruiz-Cabello, David Otaegui, Pedro Ramos-Cabrer

**Affiliations:** 1 Center for Cooperative Research in Biomaterials (CIC biomaGUNE), Basque Research and Technology Alliance (BRTA), Paseo Miramón 182, 20014 Donostia-San Sebastián, Spain; aegimendia@cicbiomagune.es (A.E.); scarregal.ciberes@cicbiomagune.es (S.C.-R.); dpadro@cicbiomagune.es (D.P.); jruizcabello@cicbiomagune.es (J.R.-C.); 2 Multiple Sclerosis Unit, Biodonostia Health Institute, 20014 Donostia-San Sebastián, Spain; inaki.osorio@biodonostia.org; 3 Spanish Network of Multiple Sclerosis, 08028 Barcelona, Spain; 4 CIBER de Enfermedades Respiratorias (CIBERES), 28029 Madrid, Spain; 5 Ikerbasque, Basque Foundation for Science, 48013 Bilbao, Spain; 6 Departamento de Química en Ciencias Farmacéuticas, Universidad Complutense de Madrid, 28040 Madrid, Spain

**Keywords:** organotypic brain cultures, myelin, demyelination, myelin-targeting liposomes, magnetic resonance imaging, molecular imaging, imaging probes

## Abstract

Ex vivo models for the noninvasive study of myelin-related diseases represent an essential tool to understand the mechanisms of diseases and develop therapies against them. Herein, we assessed the potential of multimodal imaging traceable myelin-targeting liposomes to quantify myelin in organotypic cultures. **Methods:** MRI testing was used to image mouse cerebellar tissue sections and organotypic cultures. Demyelination was induced by lysolecithin treatment. Myelin-targeting liposomes were synthetized and characterized, and their capacity to quantify myelin was tested by fluorescence imaging. **Results:** Imaging of freshly excised tissue sections ranging from 300 µm to 1 mm in thickness was achieved with good contrast between white (WM) and gray matter (GM) using T2w MRI. The typical loss of stiffness, WM structures, and thickness of organotypic cultures required the use of diffusion-weighted methods. Designed myelin-targeting liposomes allowed for semiquantitative detection by fluorescence, but the specificity for myelin was not consistent between assays due to the unspecific binding of liposomes. **Conclusions:** With respect to the sensitivity, imaging of brain tissue sections and organotypic cultures by MRI is feasible, and myelin-targeting nanosystems are a promising solution to quantify myelin ex vivo. With respect to specificity, fine tuning of the probe is required. Lipid-based systems may not be suitable for this goal, due to unspecific binding to tissues.

## 1. Introduction

Myelin is an essential element of the central nervous system (CNS) and is implicated in many neurological diseases. The study of myelin loss (demyelination) and regeneration (remyelination) is of paramount importance for a better understanding of such processes, as well as for the design of therapeutic strategies for myelin-related diseases. The development of suitable in vitro models that allow for reliable myelin quantification in the study of de- and remyelination processes is a very important and not yet fully resolved issue that may help to boost the development of novel therapies for neurological diseases [1].

Indeed, the use of in vitro models may facilitate the obtaining of faster results, more controllable and reproducible experimental conditions, and more statistically powerful results than in vivo models. The possibility of isolating the target subject of study from concomitant confounding effects present in the complexity of in vivo settings (e.g., avoiding the effects of an immune response) is an additional important feature of in vitro models.

The development of an in vitro model is a complex task that requires not only the definition of a suitable system that mimics the relevant biological aspects that are expected to be replicated, but the definition of proper experimental techniques that allow for observing and, ideally, quantifying the phenomena under study.

In general, in vitro models may be constructed with increasing degrees of complexity, starting from specific cell-type monocultures and scaling to complex co-cultures (including the physical separation of cells by permeable membranes, flowing fluids, etc.), organoids, perfused excised organs or tissues, and 3D bioprinted structures. Despite multiple in vitro models having been successfully reported in the literature for the study of the central nervous system (CNS) [2], for myelin-related diseases, it is important to replicate the complex multifaceted nature of the brain, with strong interactions among its components, a task that is difficult to achieve using just cell cultures. In this case, ex vivo excised brain sections transformed into organotypic brain cultures (OBCs) have been proposed as a more realistic setting, in which glia closely interact with neuronal axons in a three-dimensional space [3]. Thus, OBCs have become a simplified way to study de- and remyelination processes and are generally considered a suitable tool for the evaluation of therapies against myelin-related diseases [4].

However, the quantification of myelin in organotypic cultures—not a trivial issue—has traditionally been tackled by invasive experimental methods such as RT-PCR, Western blot, or immunofluorescence [5]. Each of the mentioned techniques has advantages and disadvantages that encourage the search for robust; universal; affordable; and, above all, noninvasive experimental techniques that allow for the quantification of myelin in a longitudinal manner. Thus, the identified disadvantages include: (1) indirectness. For example, RT-PCR enables the quantification of myelin-related genes but does not provide a direct measure of myelin content. (2) Limited sensitivity. For example, Western blot can provide a semiquantitative measure of myelin content but is not sensitive enough to detect the myelin content of single tissue slices. (3) Limited quantifiability. For example, immunofluorescence is based on the separate staining of axons and myelin and co-localization of imaging channels—a challenging way to reliably quantify myelin. Other methods, such as long-term transgene-induced fluorescence live imaging, have also been performed in organotypic cultures of mice [6], but no single experimental technique has shown sufficient simplicity, accuracy, and reproducibility to assess myelin content in OBCs in a robust manner.

In this context, we have explored the possibility of using functional biomaterials for the quantitation of myelin content in OBCs by magnetic resonance imaging (MRI), which has the great advantage of being noninvasive and therefore enabling longitudinal studies in the same cultures. Despite MRI having been widely used for the quantification of myelin in clinical and preclinical in vivo studies, with several MR imaging biomarkers of myelin, such as T2-weighted MR signal or radial diffusivity [7], the use of this imaging technique for the study of organotypic cultures represents a huge challenge, due to several factors related to the changes that brain tissue samples experience when evolving into organotypic cultures (including the loss of white matter or gray matter structuring, loss of stiffness, and thinning of tissue samples, which spread out on the surface of the supporting membrane over time) [8].

We believe that the use of molecular imaging approaches, including the use of targeting imaging probes that help to increase the sensitivity of detection, may offer an opportunity to overcome such challenges; thus, in this work, we describe our attempts to achieve an experimental protocol for the MRI-based noninvasive imaging of tissue sections and organotypic cultures, and to develop myelin-specific imaging nanoprobes that enable the quantification of myelin content in organotypic cultures from the mouse cerebellum.

The development of nanomaterials that act as imaging probes for MRI is an active field of research, and examples can be found in the literature [9,10,11]. In the past, we developed liposome-based imaging probes [12], which may be suitable for the detection of myelin in OBCs.

The results demonstrate that, although it is feasible to image organotypic brain cultures (OBCs) by MRI, and molecular imaging enables the quantification of myelin in ex vivo models, the design of the functional imaging probe is an especially critical issue that requires further development to ensure the sufficient sensitivity for myelin, avoiding specificity-related issues.

## 2. Materials and Methods

All experiments involving the use of research animals have been performed according to the appliable legislation (European Union Directive 2010/63/EU on the protection of animals used for scientific purposes) and were approved by our Institutional Animal Care and Use Committee (IACUC) and by the local authorities (*Diputación Foral de Guipuzcoa* and our institutional ethics committee, with license number CEEA17_002). Studies were conducted on three different types of samples.

### 2.1. Brain Tissue Sections

C57BL/6JRj mice (Janvier Labs, Le Genest-Saint-Isle, France) were sacrificed at postnatal day 7–12, and their cerebellums were sliced to different thicknesses (typically 1.9 mm, 0.8 mm, 0.5 mm, 0.3 mm, or 0.1 mm) and fixed in paraformaldehyde (10%) for 40 min. Next, the brain sections were washed 2× (for around 10 min) with phosphate-buffered saline (PBS) and immersed in 2% low melting point agarose (Ref: A9539; Sigma-Aldrich, St. Louis, MO, USA) in a 50 mL Falcon tube for imaging. The possibility of performing multiplanar MR imaging allows for scanning sets of several tissue sections in a single imaging experiment by piling the sections inside the agar gels.

### 2.2. Thick Organotypic Cultures

C57BL/6JRj mice (Janvier Labs, Le Genest-Saint-Isle, France) were sacrificed at postnatal day 10–12 and their cerebellums immediately extracted and placed in an organotypic culture medium prepared with 24 mL 2-mercaptoethanol (BME) (Ref. 41010; Thermo Fisher Scientific, Waltham, MA, USA), 24% Hanks’ balanced salt solution (HBSS) (REF. 24020091; Thermo Fisher Scientific), 24% horse serum (Ref. 26050088; Thermo Fisher Scientific), 0.125% glutamine (Ref. 25030024; Invitrogen, Carlsbad, CA, USA), 1% antimycotic and antibiotic (Ref. A5955; Sigma-Aldrich), and 3.5% glucose (Ref. A1422, Panreac Química SLU, Barcelona, Spain), for a total solution volume of 50 mL.

**Figure 1:**
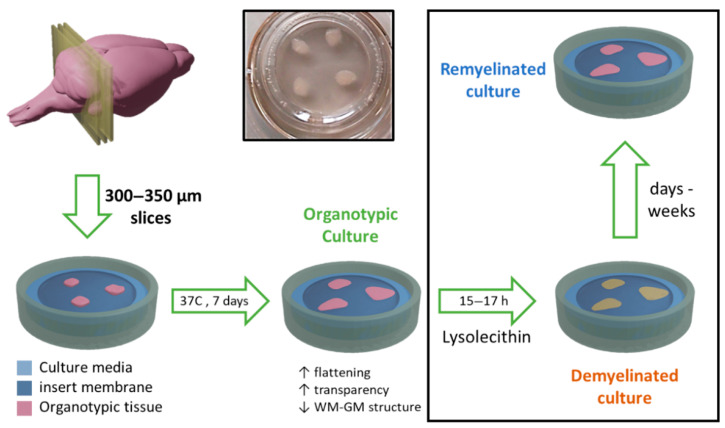
Preparation of cerebellar organotypic brain cultures from tissue sections. Postnatal (7–12 days) mouse brains were extracted and sliced into 300–350 µm sections. Slices were cultured for one week on a permeable membrane in contact with the medium. Optionally, lysolecithin may be used to induce demyelination. Remyelination may take place spontaneously or may be promoted by therapeutic intervention. (WM: white matter, GM: gray matter).

Following this procedure, the cerebellums were sliced into 750–800 µm sagittal sections using a McIlwain tissue chopper (World Precision Instruments Ltd., Hitchin, UK). Sections were separated and placed on a Millicell Cell Culture Insert membrane (Ref. PCIM ORG 50, Millipore Corp., Burlington, MA, USA) on a P6 plate and incubated for seven days in an organotypic culture medium (as described above) at 37 °C and 5% CO_2_ (Figure 1).

For imaging studies, cultures were fixed with 4% paraformaldehyde for 40 min and kept in PBS (0.05% sodium azide) at 4 °C until use. Organotypic cultures were embedded in 2% agarose before MRI scanning. For this purpose, the support membrane of the culture insert was gently cropped around the organotypic tissue with a scalpel.

### 2.3. Thin Organotypic Cultures

Thin organotypic culture samples were prepared as described in the previous section, but with slices of 350 µm thickness, which represents the desired target for our experimental ex vivo assay (see Section 3).

When required, demyelination was induced in these cultures after one week of culturing by exposing OBCs for 15–17 h to a medium containing 0.5 mg/mL lysolecithin (Ref: L4129; Sigma).

### 2.4. Myelin-Targeting Liposomes

MRI-traceable myelin-targeting liposomes were prepared by the lipid film hydration and extrusion method [12], using a mixture of lipids composed of 1,2-distearoyl-sn-glycero-3-phosphocholine (DSPC), at molar fraction x = 0.6; 1,2-distearoyl-sn-glycero-3-phosphoethanolamine-N-[maleimide(polyethylene glycol)-2000] (ammonium salt) (PEG-DSPE), at a molar fraction of x = 0.025; (diethylenetriaminepentaacetic acid)-bis(stearylamide) (gadolinium salt) (Gd-DTPA-BSA), at a molar fraction of x = 0.017; and cholesterol (Chol), at molar fraction x = 0.333. All liposome components (obtained from Avanti Polar Lipids, Alabaster, AL, USA) were dissolved and mixed in a chloroform–methanol mixture (6:1). After solution, a lipid film was formed by the evaporation of the organic solvent on a rotavapor (high vacuum at 30 °C) and drying under nitrogen flow for 2 h. The lipid film was then rehydrated with 7 mL of HPLC-grade water (Ref. W/0106/17; Thermo Fisher Scientific) at 65 °C. Rehydrated liposomes were extruded 14 times at 65 °C through polycarbonate membrane filters (Whatman PLC, Maidstone, UK) using consecutive decreasing pore sizes of 400 nm (×2), 200 nm (×4), and 80 nm (×8). After extrusion, liposomes were stored at 4 °C and characterized for lipid content by the Rouser method [13], measuring size, and z-potential by dynamic light scattering, using a Malvern z-Sizer system (Malvern Panalytical, Malvern, UK), as described elsewhere [12].

Liposomes were designed as multimodal imaging probes detectable by magnetic resonance imaging and fluorescence microscopy. Thus, gadolinium ions (Gd^3+^) responsible for the generation of T1-weighted MRI contrast were complexed with the lipid DTPA-BSA(Gd) (Ref. 791268P, Avanti Polar Lipids), used as a constitutive element of the liposome membrane. It was possible to tailor the magnetic properties of liposomes to obtain the best performance from the MRI by modifying the DTPA-BSA(Gd) percentage during the lipid film formation. Four different molar fractions of Gd-BSA lipid were assayed (x = 0.017, 0.033, 0.100, 0.133) to achieve the highest T1-induced contrast. In parallel, the fluorescent dye DiOC18(3) (3,3′-dioctadecyloxacarbocyanine perchlorate, Ref. D275; Thermo Fisher Scientific) was added (300 μL of a 1 mg/mL solution) to the formulation in the organic phase before a lipid film was formed.

Liposomes were designed to specifically target myelin. Thus, the maleimide groups present in the surface of liposomes were conjugated with the antimyelin basic protein antibody (Ref. Ab62631; Abcam, Cambridge, UK), substituted by anti-IgG protein antibody in control liposomes (Ref. ab18447; Abcam). For conjugation, the antibody was preactivated by mixing it in SATA solution (1:80 mol/mol). Afterwards, the SATA–antibody solution was added to the liposome solution in a vial (50 µg of protein per 1 µmol of lipids), and the mix was kept overnight at 4 °C under N_2_ atmosphere. Uncoupled protein was removed from the liposome solution by centrifugation (65,000 rpm, 45 min) and resuspension of the pellet containing the liposomes in HEPES-buffered saline (HBS, pH 7.4). Transmission electron cryomicroscopy (CryoTEM), dynamic light scattering (DLS), and inductively coupled plasma mass spectrometry (ICP-MS) were used for the physicochemical characterization. A Bradford Assay (Ref. B6916; Sigma-Aldrich) was used to verify that antibodies were attached to the surface of the liposomes. Relaxometry properties were measured in a Bruker Minispec MQ60 (Bruker Biospin GmbH, Ettlingen, Germany) contrast agent analyzer at 1.5 T and 37 °C.

### 2.5. Molecular Recognition of Myelin by Targeting Liposomes

Cell culture inserts were prepared, each of them with three thin organotypic slices lying on the surface of the membrane (Figure 1). After a one-week incubation, cultures were demyelinated by adding lysolecithin (Ref. L4129; Sigma-Aldrich) at a concentration of 0.5 mg/mL to the culture medium, for a period of 15–17 h (Figure 1). Removing this chemical from the organotypic culture media triggers the spontaneous remyelination of organotypic cultures, a process that may take from days to weeks, and can be enhanced by therapeutic interventions (Figure 1). Control cultures were also prepared in the same way but without exposing them to lysolecithin.

For myelin recognition by liposomes, cultures (control, demyelinated, and remyelinated) were fixed with 4% paraformaldehyde for 40 min and washed with DPBS. Tissue was blocked with a solution composed of DPBS, 0.5% Triton (Ref. T8787; Sigma-Aldrich), and 10% goat serum (Ref. G9023; Sigma-Aldrich) for 1 h at room temperature. A volume of 350 µL of liposomes was added and samples were incubated overnight at 4 °C. Then, samples were washed with 0.1% Triton in DPBS and stained with Hoechst (Ref: B2261; Sigma) 10% in DPBS for 10 min.

### 2.6. Imaging Studies

Fluorescence images were acquired using a Nikon Eclipse 80i digital microscope (Nikon Instruments, Melville, NY, USA) and processed using NIS elements AR 3.2 software (Nikon).

MRI studies were conducted at 7 Tesla using a Bruker Biospec 70/30 USR MRI system (Bruker Biospin GmbH, Ettlingen, Germany). Images of brain tissue slices and thin organotypic cultures were acquired with spatial resolutions ranging from 25 × 25 × 25 µm^3^ to 100 × 100 × 500 µm^3^. Different T2-weighted imaging sequences were tested (RARE, MSME, and FSE) with echo times ranging from 10 to 70 ms and repetition times ranging from 2000 to 7000 ms.

For thin organotypic cultures, diffusion-weighted imaging was also conducted with a spin-echo diffusion-weighted imaging (DWI) sequence, using the following parameters: b-value = 1500 s/mm^2^; gradient pulse durations δ = 6 ms and spacing Δ = 14 ms; TR = 2820 ms; slice thickness = 250 µm.

Imaging of thick organotypic slices was performed with a turboRARE sequence with the following parameters: TR = 2000 ms; RARE factor = 10; effective echo time TE = 70 ms; field-of-view (FOV) = 10 mm × 10 mm; image matrix = 400 × 400 points; 24 slices with a thickness of 0.3 mm.

Image analytics were conducted using the NIH software FIJI [14]. For the analysis of the images, signal-to-noise ratios (SNR) were calculated for white and gray matter at different tissue thicknesses and both spatial resolutions (Table 1). SNR was defined as the mean signal intensity in a region of interest (ROI) of the tissue under observation, divided by the standard deviation of the noise, obtained from a ROI at the background, outside of the object of interest (SNR = 0.66 × mean_tissue_/SD_background_). The contrast -to-noise ratio (CNR) between gray and white matter was obtained as the difference of the mean of each tissue divided by the standard deviation of the background (CNR = 0.66 × [mean_gm_ − mean_wm_]/SD_background_).

## 3. Results and Discussion

As discussed in the introduction, the development of remyelination therapies requires experimental models and techniques that enable the evaluation of the effect of a given therapy. Both in vitro and in vivo models have been proposed in the literature, with growing interest in mixed models such as the use of ex vivo organotypic brain cultures (OBCs), in which the advantages of in vitro systems such as reproducibility, homogeneity, high-throughput capability, etc. can be applied to brain tissue samples with a more complex structure than a culture. Thus, OBCs are being exploited for the study of remyelination therapies. However, a reliable and noninvasive technique thar allows for the longitudinal assessment of myelin content in organotypic cultures is still an unresolved issue [4,15] that we have attempted to tackle by following a sequential reductionistic approach, described below.

### 3.1. Imaging Thick Tissue Sections

First, we tested the capability of MRI to image thick and large portions of cerebellar tissue, fixated in formalin briefly after excision from mouse brains. This does not represent a proper model of organotypic cultures per se but allowed us to establish a starting point from manageable samples in the MRI scanner, assess the limits of the achievable sensitivity of detection (signal-to-noise and contrast-to-noise ratios), and find the working limits of image slice thickness and spatial resolution affordable for our MRI scanner.

**Figure 2:**
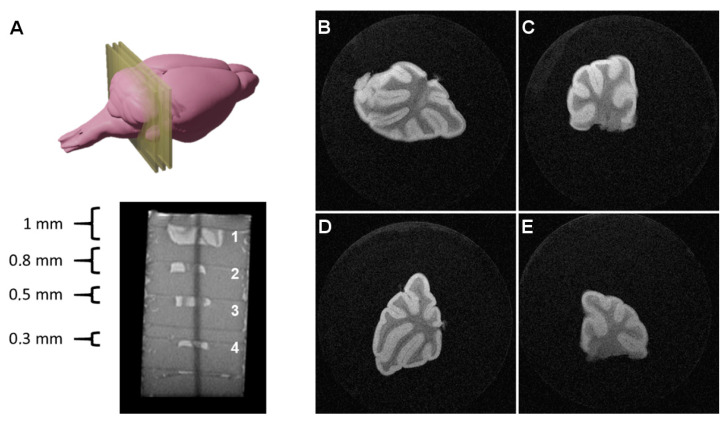
MR imaging of thick tissue slices: (**A**) mouse cerebellum tissue slices excised at different thicknesses and stacked in agar (sagittal MR image); (**B**–**E**) coronal MRI 2D images (300 µm slice thickness) acquired at two in-plane resolutions. White matter structures (hypointense on T2w imaging) are clearly distinguishable from gray matter.

Thus, several tissue sections of different thicknesses (0.3, 0.5, 0.8, and 1 mm) were prepared, imbibed in agar gels, and scanned with multiple acquisition sequences, in order to image such thick tissue slices, optimizing the balance between imaging time, SNR, CNR, spatial resolution, and image quality (Figure 2).

The best results were achieved using a RARE (Rapid Acquisition with Relaxation Enhancement, also referred to as Fast Spin Echo or Turbo Spin Echo) sequence with a RARE factor of 8, an echo time of TE = 16 ms (effective echo time of TE_effective_ = 64 ms); FOV = 1.28 × 1.28 cm^2^; image matrix = 512 × 512 (in-plane resolution: 25 × 25 µm^2^), slice thickness = 300 µm; *N* = 20 averages; total scanning time = 2 h 4 min.

The images presented in Figure 2, where white matter appears as hypointense and gray matter as hyperintense due to the T2 contrast, were used to calculate SNR values, calculated as the ratio between the mean signal in a region of interest including 75% of the tissue and the standard deviation of the noise, collected from a ROI of the background, far from the object and avoiding potential influences of readout and phase encoding ghosting, multiplying this ratio by a factor of 0.66, as described in Goerner et al. [16]. The results of these calculations are presented in Table 1.

As shown in Table 1, for this set of samples, the tissue thickness has little influence on the SNR and CNR values, presenting no significant differences for acquisitions in which the imaging slice thickness is the same, and completely covers the tissue section, in this way avoiding partial volume effects. In fact, slightly inferior SNR values were observed for a tissue sample of 300 µm in thickness, matching the imaging slice thickness, representing the limit at which partial volume effects start to show up, since it is difficult to have a completely flat tissue section in the agar perfectly aligned with the imaging slice to completely avoid those effects. Imaging of thinner tissue slices will be discussed in the next section.

### 3.2. Imaging Thick Organotypic Cultures

The tissue samples described in Section 3.1 were fixed and embedded in agar just after excision from the mice cerebellums. In a second set of experiments, we went one step forward by culturing brain slices for a period of 7–12 days (as is usually done when preparing OBCs), the period during which a loss of tissue thickness and stiffness is expected. Thus, we cultured a series of 750 µm thick brain slices, as described in Section 2 and represented in Figure 1.

**Figure 3:**
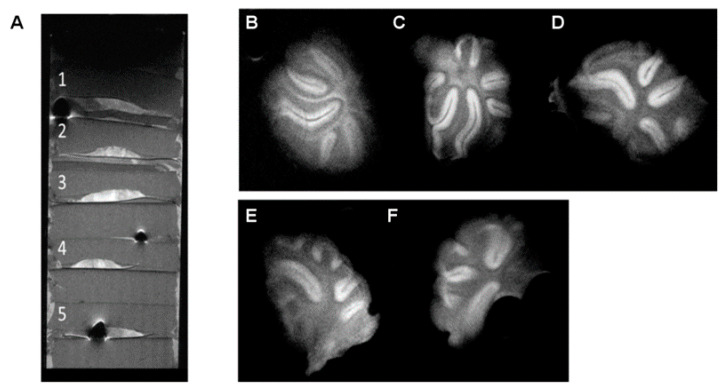
MR imaging of thick organotypic cultures: (**A**) sagittal MR image of five mouse cerebellum organotypic cultures stacked in agar (sagittal MR image); (**B**–**F**) coronal MRI 2D images (300 µm slice thickness) of the organotypic slices. The white matter–gray matter limit blurred as the cultured tissues evolved.

As expected for tissue samples evolving into organotypic cultures, incubation affected the tissue thickness, resulting in approximately half-thickness slices (≈300 µm, which corresponds to the minimum tissue sample thickness studied in Section 3.1). Incubation also affected the tissue integrity, showing more diffuse limits between white matter and gray matter, appearing somewhat blurred in MR images (Figure 3).

Only the results for the optimized MR imaging sequence and target spatial resolution are presented here. The mean calculated SNR for this cultured tissue was 62.7 for gray matter and 38.9 for white matter, with a CNR of 31.9 between both tissues. From these results, we can conclude that good-quality images can be obtained from these cultured tissues, where white matter is still clearly identifiable. However, the lines defining the limit between white and gray matter, as well as the external limits of the culture sections, started to look blurry and less defined when compared to those observed in the prior study with tissue sections.

This experimental setup is closer but still does not represent proper organotypic cultures. Typical organotypic cultures were prepared from tissue sections with a maximum thickness of 300–350 µm. When thicker tissue sections are cultured using a setup such as the one shown in Figure 1 (like the 750 μm sections used here), the permeation of nutrients and oxygen to the inner parts of the tissues is compromised, usually leading to cell loss and tissue necrosis [3]. Even though attempts have been carried out to culture organotypic thick slices [17], they have not been successfully adapted for the study of de- and remyelination processes thus far.

### 3.3. Imaging Thin Organotypic Cultures

**Figure 4:**
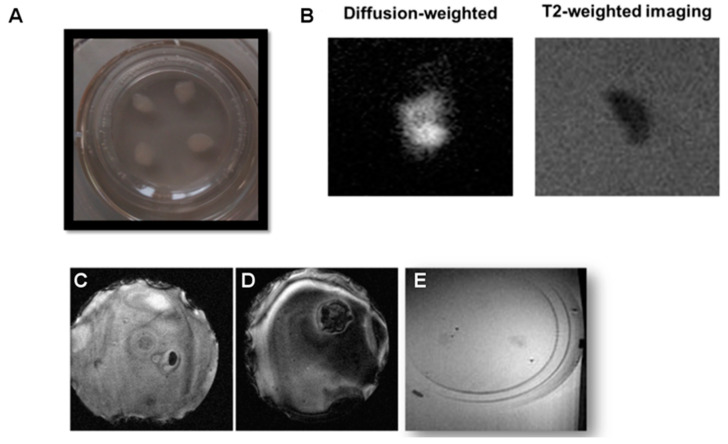
MR imaging of thin organotypic slices. (**A**) A photograph of organotypic slices in culture. (**B**) Diffusion-weighted vs. T2-weighted imaging of an organotypic slice. (**C**–**E**) Different imaging artifacts observed when imaging thin tissue slices ((**C**) air bubble; (**D**) misalignment of the supporting membrane with imaging plane; (**E**) loss of CNR due to partial volume effects).

In a final set of experiments, tissue sections of 350 µm were excised and cultured for 7–12 days, obtaining in this way conventional organotypic cultures that are typically prepared from slices of such thickness, and reaching values of around 100 µm at the end of the incubation period. It should be borne in mind that the flattening and increased transparency of the tissue sections over time (Figure 4A) are actually indicators of tissue health and survival [3]. In this sense, using mice at postnatal day 12 resulted in a firmer structure compared to younger cultures, facilitating MRI imaging and, at the same time, enabling tissue survival.

Imaging of thin organotypic slices is highly challenging and not satisfactory. Many problems are encountered when imaging these cultures. Firstly, the excised tissues evolve into a sort of slick mass after culturing, hardly attached to the supporting membrane, which is a rigid structure. Positioning of this rigid structure in agar gels is hard to perform while avoiding the formation of small air bubbles, which generate strongly susceptible artifacts that spoil the image (Figure 4C). On the other hand, the porous film supporting the culture can hardly be kept flat in the agar, complicating the orientation of the imaging planes parallel to the thin tissue sample, creating in this way large “black shadows” on the image due to the lack of signal of the supporting membrane, which contains no protons to image (Figure 4D). Of course, one can consider removing the tissue from the membrane for MR imaging, but this will affect OBCs’ viability and hamper the recovery of the tissue for repeated imaging if one likes to follow a longitudinal approach.

In addition, even when the aforementioned distorting effects can be avoided, it is hard to distinguish tissues from the surrounding agar on T1, T2, or T2 * weighted images, since the contrast between the tissue and the background agar is seriously affected by partial volume effects as a consequence of the reduced thickness of the tissues at the moment of imaging (tissues spread on the surface of the membrane during culturing, from the original 350 µm to <100 µm on the imaging day). The minimal imaging slice thickness is determined by hardware constrictions (gradient strengths and SNR of the radiofrequency coils) and the imaging time (the thinner the slice, the higher number of averages required to achieve a high enough SNR). Our experimental conditions allowed us to acquire a minimum imaging slice thickness of 300 μm, in practice, and at this condition <1/3 of the voxel was filled with tissue, with the rest being occupied by agar. When ideal conditions were met, it was possible to obtain images from OBCs (Figure 4B,E), but with very low SNR, and with it no longer being possible to distinguish between white and gray matter.

In light of these results, we attempted a different approach for conducting diffusion weighted imaging, whereby the signal arising from water molecules in the agar gel is suppressed by diffusional gradients (Figure 4B). In this case, it was possible to identify organotypic cultures at high SNR (SNR > 45). However, the evolution of the tissue into a smooth blob comes with a loss of internal structures, and white matter is no longer distinguishable from gray matter (Figure 4B). In this sense, it is not possible to quantify myelin content, which was our original objective. Therefore, we concluded that conventional MRI, in any of its modalities, is not an ideal experimental technique to perform the longitudinal quantification of myelin in organotypic cultures, and thus a different experimental approach was developed.

### 3.4. Molecular Imaging Recognition of Myelin by Targeting Liposomes

As we have mentioned, sensing technology uses sensors to detect a physical, chemical, or biological property with an experimental technique, and convert it into a readable signal that allows for quantification. The development of nanomaterials acting as imaging probes for MRI is an active field of research [9,10,11]. In the past, we developed liposome-based imaging probes [12] that may be suitable for the detection of myelin in OBCs.

**Figure 5:**
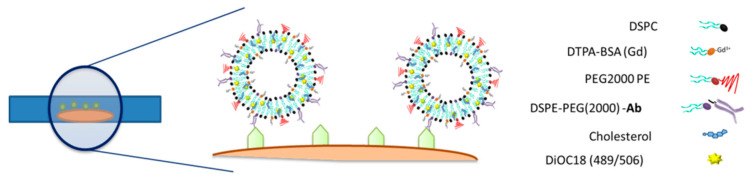
Molecular imaging strategy for the quantification of myelin in organotypic cultures. Antimyelin stealth liposomes doped with gadolinium and Dioc18 were incubated with the cultured tissue for later detection with imaging techniques (see Section 2 for the full names of the abbreviated chemicals in the figure).

Liposomes doped with imaging probes (gadolinium for MRI detection and a fluorescence probe for microscopy) and decorated with antimyelin basic protein antibody (anti-MBP) were prepared (as well as liposomes decorated with unspecific anti-IgG protein as a control) and incubated with thin organotypic tissues (Figure 5), as described in Section 2.

**Figure 6:**
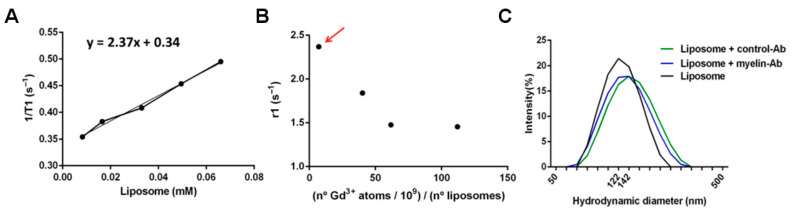
Characterization of liposomes. (**A**) Relaxation rate vs. concentration plots. (**B**) Longitudinal relaxivity vs. load of gadolinium plot, of prepared liposomes. (**C**) Hydrodynamic diameters of liposomes, as determined by DLS.

Different liposomal compositions were prepared to optimize the load of MR contrast agent, in order to achieve higher sensitivity of detection. Thus, up to four formulations of liposomes were synthesized, with increasing amounts of gadolinium (7.1 × 10^9^, 40.2 × 10^9^, 61.7 × 10^9^, and 112 × 10^9^ Gd atoms per liposome unit, as measured by inductively coupled plasma mass spectrometry (ICP-MS), and characterized by measuring their longitudinal relaxivity (r1), calculated as the slope of plots of the relaxation rate (R1 = 1/T1, where T1 is the longitudinal relaxation time) of solutions of different concentration of those liposomes versus the concentration of liposomes (Figure 6).

An important aspect of the development of imaging sensors is the optimization of the payload of imaging probes (gadolinium content per liposome) since, in the case of MRI, it has been demonstrated that an excessive payload of this lanthanide in the probe could quench the T1 effect and reduce the sensitivity of detection and image contrast [18]. Thus, several formulations were prepared, containing different amounts of gadolinium per liposome unit. Then, the longitudinal magnetic relaxivity of each formulation was calculated as the slope of plots of measured relaxation rates (the inverse of the relaxation times: R1 = 1/T1) versus the concentration of gadolinium (Figure 6A), as determined by mass spectrometry [18]. The optimized load of gadolinium in liposomes (in terms of higher longitudinal relaxivity) was achieved for a load of 7.1 × 10^9^ atoms of gadolinium atoms per liposome (corresponding to a molar fraction of 0.017 Gd lipids in the formulation), resulting in a longitudinal relaxivity of 2.17 mM^−1^s^−1^, with respect to the concentration of liposomes. As we anticipated, higher loads of gadolinium resulted in a partial quenching of T1 contrast (Figure 6B).

The decoration of the surface of the naïve liposomes with antimyelin protein or IgG (control) protein attached to polyethylene (PEG) chains has no significant effects on liposomes’ sizes, as determined by dynamic light-scattering studies (Figure 6C; the mean liposome hydrodynamic diameters were naïve: 124.0 ± 1.5 nm; anti-IgG: 133.9 ± 1.5 nm; anti-myelin: 124.7 ± 1.5 nm), which is an important feature to prevent alterations in the behavior of liposomes as sensing devices.

**Figure 7:**
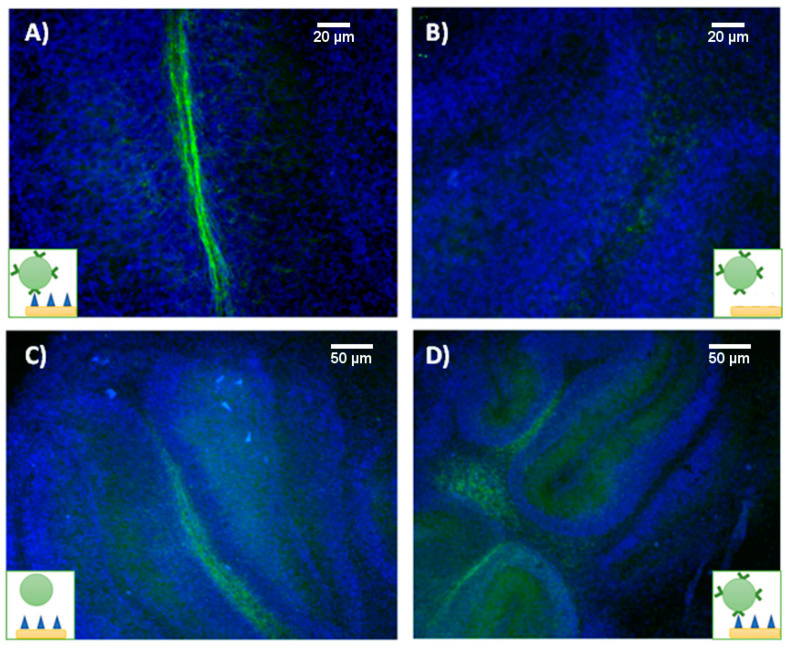
Fluorescence imaging of tissue sections (blue for cell nuclei and green from DIOC18 fluorescent liposomes) incubated with targeting liposomes. (**A**) Healthy and (**B**) demyelinated tissues, incubated with fluorescent anti-MBP liposomes. (**C**) Healthy tissues incubated with control anti-IgG and (**D**) with antimyelin basic protein liposomes.

Myelin-targeting liposomes were incubated with both myelinated and demyelinated organotypic cultures and imaged by fluorescence microscopy after washing unbound liposomes from the culture media. In panels A and B of Figure 7, we present fluorescence images of myelinated and demyelinated tissues, on which cell nuclei are stained blue, incubated with antimyelin green fluorescent liposomes, showing that sensing of myelin sheaths is feasible using our experimental approach.

Imaging studies showed that green fluorescent MBP-targeting liposomes can specifically bind to myelin sheaths in healthy organotypic cultures (Figure 7A), while no visible attachment was observed when demyelinated cultures were cultured with the liposomes (Figure 7B). Unfortunately, both nontargeted (IgG control) and myelin-targeted liposomes experienced nonspecific binding (Figure 7C,D, respectively) to tissue in OBCs. Despite the different incubation strategies followed (varying incubation times and liposome concentration in the media), we were not able to avoid this phenomenon.

Certainly, it has been described that liposomes have an affinity for biological tissues, in general, due to electrostatic and nonspecific hydrophobic forces [19]. In the case of myelin sheaths, their lipidic nature increases even further their affinity for liposomes, presenting slightly increased fluorescence compared to the surrounding tissue, but the general unspecific binding of liposomes to all tissue, irrespective to the presence of targeting antibodies, discourages their use as sensing probes for the molecular recognition of myelin in OBCs.

Overall, our results have provided proof-of-concept that molecular recognition via imaging-driven sensing studies is feasible and a good approach for the detection and quantification of myelin in organotypic cultures. However, liposomes do not seem to be an ideal molecular platform to perform this task. The development of a different sort of nanomaterial with no affinity for biological tissues is a must, but is a complex task that requires fine-tuning of the composition, structural and physicochemical properties, as well as the study of the biological compatibility (cytotoxicity) and optimization of imaging properties, which is outside of the scope of this work. Work along this line is currently in progress in our laboratories.

A limitation of this study is that we do not report the MRI detection of myelin in OBCs through the use of prepared liposomes. Due to the much higher sensitivity of detection of fluorescence imaging versus MRI, we performed tests of specificity with this technique. Since multimodal imaging liposomes were nonspecific for myelin sensing, we decided not to continue MRI studies with them and instead will wait until an optimized nanomaterial is available. We will continue with the search for alternative materials that may be effective for the specific labeling of myelin in organotypic brain cultures.

## 4. Conclusions

The development of experimental protocols for the noninvasive in vitro characterization of myelin in cultures may represent a breakthrough for the performance of high-throughput studies of treatments, in highly reproducible experimental conditions. In this work, we have shown that the MRI imaging of freshly excised and fixed brain slices is feasible with high SNR and CNR, demonstrating the potential of this technique for the quantification of myelin, but such systems are inadequate as ex vivo models, since the preservation of cells in the interior of thick sections in culture is highly compromised by the limited diffusion of nutrients and oxygen through them.

We have also shown that thin organotypic cultures are inherently problematic for imaging and quantification by MRI and that, alternatively, sensing of myelin by molecular recognition with multimodal imaging nanomaterials can be a suitable experimental strategy, providing proof-of-concept for this approach, but we have not yet succeeded in finding a suitable molecular platform to achieve the required level of specificity required for this task, since liposomes have high unspecific affinity for biological tissues in general. Ongoing research in this line will tell us if this is a good approach for obtaining experimental ex vivo tools for the longitudinal study of demyelination and remyelination processes.

## Figures and Tables

**Table 1:** Signal-to-noise and contrast-to-noise values for MRI images of thick tissue slices. SNR: signal-to-noise ratio; CNR: contrast-to-noise ratio; GM: gray matter; WM: white matter.

Tissue Thickness (mm)	25 × 25 µm^2^ Resolution		
	SNR_GM_	SNR_WM_	CNR_GM-WM_
1.0	6.7	4.0	2.7
0.8	7.4	4.4	2.9
0.5	6.0	4.0	2.0
0.3	5.6	3.5	2.1

## Data Availability

Raw imaging data will be available from the corresponding author P.R.-C. on reasonable demand.

## References

[1] Osorio-Querejeta I., Sáenz-Cuesta M., Muñoz-Culla M., Otaegui D. (2017). Models for Studying Myelination, Demyelination and Remyelination. Neuromol. Med..

[2] O’Rourke C., Lee-Reeves C., Drake R.A., Cameron G.W., Loughlin A.J., Phillips J.B. (2017). Adapting tissue-engineered in vitro CNS models for high-throughput study of neurodegeneration. J. Tissue Eng..

[3] Humpel C. (2015). Organotypic brain slice cultures: A review. Neuroscience.

[4] Tan G.A., Furber K.L., Thangaraj M.P., Sobchishin L., Doucette J.R., Nazarali A.J. (2018). Organotypic Cultures from the Adult CNS: A Novel Model to Study Demyelination and Remyelination Ex Vivo. Cell Mol. Neurobiol..

[5] Pang Y., Zheng B., Kimberly S.L., Cai Z., Rhodes P.G., Lin R.C. (2012). Neuron-oligodendrocyte myelination co-culture derived from embryonic rat spinal cord and cerebral cortex. Brain Behav..

[6] Gogolla N., Galimberti I., DePaola V., Caroni P. (2006). Long-term live imaging of neuronal circuits in organotypic hippocampal slice cultures. Nat. Protoc..

[7] Laule C., Vavasour I.M., Kolind S.H., Li D.K., Traboulsee T.L., Moore G.R., MacKay A.L. (2007). Magnetic resonance imaging of myelin. Neurotherapeutics.

[8] Jarjour A.A., Zhang H., Bauer N., Ffrench-Constant C., Williams A. (2012). In vitro modeling of central nervous system myelination and remyelination. Glia.

[9] Ramos-Cabrer P., Campos F. (2013). Liposomes and nanotechnology in drug development: Focus on neurological targets. Int. J. Nanomed..

[10] Ramos-Cabrer P., Campos F., Sobrino T., Castillo J. (2011). Targeting the ischemic penumbra. Stroke.

[11] Lim E.K., Jang E., Lee K., Haam S., Huh Y.M. (2013). Delivery of cancer therapeutics using nanotechnology. Pharmaceutics.

[12] Agulla J., Brea D., Campos F., Sobrino T., Argibay B., Al-Soufi W., Blanco M., Castillo J., Ramos-Cabrer P. (2013). In vivo theranostics at the peri-infarct region in cerebral ischemia. Theranostics.

[13] Rouser G., Fkeischer S., Yamamoto A. (1970). Two dimensional then layer chromatographic separation of polar lipids and determination of phospholipids by phosphorus analysis of spots. Lipids.

[14] Schindelin J., Arganda-Carreras I., Frise E., Kaynig V., Longair M., Pietzsch T., Preibisch S., Rueden C., Saalfeld S., Schmid B. (2012). Fiji: An open-source platform for biological-image analysis. Nat. Methods.

[15] Zhang H., Jarjour A.A., Boyd A., Williams A. (2011). Central nervous system remyelination in culture—A tool for multiple sclerosis research. Exp. Neurol..

[16] Goerner F.L., Clarke G.D. (2011). Measuring signal-to-noise ratio in partially parallel imaging MRI. Med. Phys..

[17] Rambani K., Vukasinovic J., Glezer A., Potter S.M. (2009). Culturing thick brain slices: An interstitial 3D microperfusion system for enhanced viability. J. Neurosci. Methods.

[18] Agulla J., Brea D., Argibay B., Novo M., Campos F., Sobrino T., Blanco M., Castillo J., Ramos-Cabrer P. (2014). Quick adjustment of imaging tracer payload, for in vivo applications of theranostic nanostructures in the brain. Nanomedicine.

[19] Akbarzadeh A., Rezaei-Sadabady R., Davaran S., Joo S.W., Zarghami N., Hanifehpour Y., Samiei M., Kouhi M., Nejati-Koshki K. (2013). Liposome: Classification, preparation, and applications. Nanoscale Res. Lett..

